# Perceived benefits and limitations of remote decision-making support for ambulance clinicians in a single NHS trust

**DOI:** 10.29045/14784726.2025.3.9.4.1

**Published:** 2025-03-01

**Authors:** Peter Eaton-Williams

**Affiliations:** South East Coast Ambulance Service NHS Foundation Trust ORCID iD: https://orcid.org/0000-0001-5664-3329

**Keywords:** emergency medical services, telemedicine

## Abstract

**Introduction:**

Remote decision-making support (RDMS) systems for on-scene ambulance clinicians aim to improve patient safety, avoid unnecessary admissions and promote appropriate referrals. In the relative absence of previous research, this qualitative study explored the perceived benefits and limitations of a well-established RDMS system in a single NHS ambulance trust. The system described involves advanced paramedic practitioners (APPs) supporting colleagues via an emergency-crew advice telephone line (ECAL).

**Methods:**

Internally circulated invitations resulted in a convenience sample of 27 participants attending online meetings for data collection. Eight meetings, with a mean duration of 56 minutes, were recorded and anonymised during transcription. A critical realist, experiential approach to thematic analysis was employed on transcripts to produce findings.

**Results:**

Participants reported various patterns of engagement with ECALs, but experienced paramedics were the least involved. ECALs were perceived to benefit patient safety and clinical development, although their influence on appropriate care delivery was considered to be more limited. The information systems, capacity and capability of community care pathways varied considerably across the region, hindering urgent care navigation. Additionally, a cultural shift to normalise collaborative decision making was required, which might be enabled by more proactive intervention, but only if ECAL interactions sustained trust in their effectiveness. Some participants had experience of initiatives where co-located community and emergency department clinicians augmented RDMS provision and perceived that this addressed many of the limitations identified.

**Conclusion:**

This study suggests that RDMS is perceived as beneficial to patient safety and appropriate care delivery, and that APPs who are familiar with their region and with the clinicians on scene are well suited to provide this support. Collaborative decision making requires honest and open interaction to be effective and needs to be more widely accepted as standard clinical practice. Improving the consistency and interoperability of community care pathways will maximise their value, and inter-professional collaboration may facilitate this.

## Introduction

To safely reduce avoidable conveyances to emergency departments (EDs), NHS ambulance services were tasked by national strategists to provide on-scene access to senior clinical advice for their clinicians (Association of Ambulance Chief Executives (AACE), 2020; NHS England, 2019). Historically, ambulance clinicians in the UK have worked in relative isolation compared to other healthcare professionals, receiving little decision-making support, and those less experienced may have been more likely to convey patients to the ED (Abrashkin et al., 2019; Barrett et al., 2023; O’Hara et al., 2014). Thus, collaborative decision making between professionals is promoted as an effective way to reduce clinical risk within a ‘see and treat’ response model, where clinicians attend a patient without subsequently conveying them to an ED, and to encourage the use of appropriate referrals (National Institute for Health and Care Excellence (NICE), 2018; NHS England, 2019).

South East Coast Ambulance Service NHS Foundation Trust (SECAmb) has a well-established system that provides its clinicians access to support from advanced paramedic practitioner (APP) colleagues in an urgent care hub via the emergency-crew advice telephone line (ECAL). Unregistered clinicians and newly qualified paramedics (NQPs) are required to seek support via the ECAL to discharge a patient, and all clinicians are encouraged to use it wherever they feel it may benefit patient care. This remote decision-making support (RDMS) system was involved in over 34,000 incidents in the year ending 31 March 2023 (SECAmb routine data).

Though emergency medical service RDMS may be more commonplace in other countries, literature reviews have concluded that this is an under-researched topic and that concurrent with a need for more quantitative research into the effectiveness of these systems, there is also a need for qualitative exploration of stakeholder perspectives to benefit the development of their format and function (Armour & Helmer, 2021; Janerka et al., 2023; Kim et al., 2020; NICE, 2018). Revealing differences between ‘work as imagined’ by management and ‘work as done’ by clinicians can provide valuable insights to inform the improvement of healthcare delivery systems (Rapport et al., 2018). Therefore, this study aimed to explore the perspectives of ambulance clinicians with access to RDMS, specifically those concerning the practical benefits and limitations of the system, to inform the evolution of these facilities.

## Methods

This qualitative study used online meetings to collect data and employed inductive reflective thematic analysis (University of Auckland, 2024). A critical realist standpoint was adopted, which considers things to exist independently of our understanding of them but believes that such an understanding will always be shaped by our related perceptions and experiences (Maxwell, 2012). Analysis was predominantly conducted on an experiential level, aiming to prioritise the overt content of participants’ contributions rather than attempting to interpret latent meaning (Braun & Clarke, 2022). Thus, analysis accepted narratives as a true reflection of participants’ perceptions and understandings of the topic, which in turn constitute the ‘truth’ that was sought.

Reflexive thematic analysis acknowledges the subjectivity inherent in the analytical process, and PEW solely analysed data using NVivo software. Member checking and triangulation are incompatible with this method, but it is believed that Yardley’s (2000) principles of sensitivity to context, rigour, transparency and importance are sufficiently met to demonstrate this study’s validity. PEW has more than 20 years’ experience of clinical work in the ambulance setting and has previously led qualitative analyses for published national studies in the UK. He has experience of using ECALs but not of providing them. Some participants were known to the author as colleagues, though no difference in recruitment strategy was employed, and throughout data collection and analysis the author reflected on the methods used, aiming to honestly address the study’s aims. Codes and themes were identified inductively and were revisited repeatedly, seeking to present a coherent representation of contributions. This resulted in a structure dictated by those aims, consisting of factors grouped together that were perceived by the author to describe either RDMS use or its perceived benefits and factors that either identify limitations to RDMS effectiveness or describe developments to potentially address those.

The study secured Health Research Authority (IRAS: 327074) and local governance approval before disseminating Trust-wide communications, inviting both patient-facing clinicians with access to ECALs and clinicians who provide support via ECAL to participate. Those expressing interest received participant information sheets and consent forms prior to completing availability calendars. Meetings on the Microsoft Teams application were then scheduled to permit as many currently identified participants as possible to attend. As meetings were scheduled while expressions of interest were still being collected, and as participant availability and subsequent attendance varied, sufficient numbers for a focus group were not always achieved. A script that was not tested prior to data collection, nor altered subsequent to its initiation, was used to guide conversations (Supplementary 1). Data collection continued until narratives were judged to contain sufficiently detailed perspectives that addressed all the study’s aims (Braun & Clarke, 2022). At the end of data collection and prior to analysis, transcripts were anonymised and edited by the researcher while recordings of the meetings were viewed to ensure their accuracy. Recordings were subsequently deleted, and transcripts and consent forms were stored securely on Trust servers.

## Results

Data collection took place between 4 October and 28 November 2023 and consisted of eight meetings with a mean recording duration of 56 minutes (Table 1).

**Table 1.** Perceived benefits and limitations of remote decision-making support for ambulance clinicians data collection meetings.

Meeting	Duration (mins)	Roles present
1	63	APP x 2; SNQP
2	61	APP x 5
3	60	APP; EP; NQP
4	42	APP
5	51	APP x 2
6	59	APP x 2; EP x 2; NQP
7	62	SAPP; EP
8	53	APP; SAPP; SNQP

APP – advanced paramedic practitioner; SAPP – student advanced paramedic practitioner; EP – experienced paramedic; NQP – newly qualified paramedic; SNQP – student newly qualified paramedic.

Overall, from 58 expressions of interest received, 27 participants provided data, with the number of participants in each meeting varying between one and eight. Sixteen participants were APPs or were studying for that role (SAPP), seven were experienced paramedics (EP) and four were NQPs or were studying for that role while already employed as patient-facing ambulance clinicians of a different grade (SNQP).

### Perceived benefits of RDMS

RDMS engagement varied according to the individual and to a variety of contextual factors, but some incidents were perceived to precipitate engagement more than others (Table 2). These included patients refusing proposed care, patients being reluctant to attend ED, end-of-life care, mental health issues, patients with frailty, complex medical history and/or cognitive impairment, paediatric patients, head injuries, abdominal pain, minor illness and minor injury. Participants described common ECAL use by unregistered clinicians and NQPs, as indicated by policy, but reported markedly less frequent use by experienced paramedics. Interestingly, when performing or studying for an APP role, engagement was perceived to rise again.

**Table 2.** Perceived benefits and limitations of remote decision-making support for ambulance clinicians.

Benefits	Limitations
Promotes patient safety	Technological factors
Promotes appropriate care delivery	Organisational factors
Supports clinical development	Cultural factors
Provides access to remote familiar peers	Human factors

*We still get more calls from band seven APPs than we do band sixes [experienced paramedics] and that’s, I think, just down to people identifying knowledge gaps and having a broader, wider understanding of the healthcare system and understanding that you don’t know what you don’t know.* (Participant 4)

Participants perceived that ECALs benefitted patient safety in several ways: urgent care hub interaction can enable access to patient records not available to crews; they can provide oversight to ensure that a comprehensive assessment has been completed guiding further assessments as required; and they can support consideration of a more complete set of differential diagnoses. The need for these elements varied considerably but, overall, sense checking of the risk–benefit balance of management plans was seen as a primary role.

*I would say that those people who are calling acknowledge that there’s a level of risk that they’re debating whether or not to accept, and when they are calling to ask about if there’s anything they’ve missed, they’re just trying to go through in their own minds a balance of the risk of what their management plan is.* (Participant 5)

Facilitating appropriate referrals was another perceived benefit of ECALs, but perhaps one more subjected to identified limitations. APPs have good awareness of the availability, capacities, capabilities and associated practicalities of referral to local external providers; they can also accept referrals for an APP to attend the patient.

*Certainly, with the older, frailer patients that we see, there’s a lot more understanding now that hospitals aren’t safe spaces for these patients and there are a lot more crews calling about what we can provide for them in the community and how we can go about supporting them with a community-based treatment plan.* (Participant 4)

Some participants felt that ECALs performed a beneficial feedback role for clinicians on scene, both educating where gaps in knowledge existed and corroborating good practice. Clinical development was shared with APPs providing RDMS, as they often researched conditions, discussed incidents with hub colleagues and learnt from crews’ experiences of attempted management plans.

Finally, another positive aspect of ECAL engagement was identified as the benefit of being able to communicate with someone detached from the scene, who is perhaps known to the caller and who, in addition to further education and some experience of other settings, has a good understanding of clinical practice in the ambulance setting.

*I suppose one of the benefits of being in the hub is you’re not there. So, you’ve not been involved, you kind of have that objective viewpoint.* (Participant 40)

*I’ve heard from a lot of crews that they much prefer to speak to the local hub, someone that they know and someone who knows them*. (Participant 34)

*So sometimes it’s quite nice to speak to someone who actually understands the environment that you’re working in*. (Participant 42)

Participants stressed that realisation of these identified benefits was constrained by numerous limiting factors, however.

### Perceived limitations of RDMS

Several technological limitations were highlighted by participants. Urgent care hub access to externally held patient records was not consistent across the Trust, and even with access, there were difficulties sourcing all relevant information, partly because different providers used different systems. There was also frustration that records of hub activity were not able to be directly entered onto the electronic patient care record, as this resulted in at least two records then being pertinent to an incident. Additionally, some APPs were perplexed that they could book GP practice and same-day emergency care appointments when delivering ‘hear and treat’ care (remote patient consultation), but not when they were co-ordinating care with a crew on scene.

*It’s sometimes like doing a jigsaw puzzle and missing half the pieces. So, for example, you can see that the patient saw their GP yesterday, but you can’t see what was discussed. You know, sometimes there’s information there that is almost helpful then it turns out not to be.* (Participant 4)

APP staffing levels were highlighted as a limitation, because a prompt response to an ECAL request was deemed critical to maintain engagement. Due to significant variation in acceptance criteria, local knowledge of pathways was valued and the availability of APP referrals, to physically attend patients, was also important. Some APPs questioned the level of support they had to remotely supervise colleagues to provide care outside of their scope of practice if it was in the patient’s best interest. Additionally, participants stressed that RDMS benefits cannot be realised if crews are not engaging with ECAL, and that currently organisational oversight and management of individuals’ engagement was minimal. Proactive approaches to intervention in incidents and routine appraisal of engagement were suggested.

*Obviously if you chat to the local APP, then they know the local pathways better than the APP from [place name] would for example. Unless I’m in [place name] of course, in which case I’d definitely call the local [place name] APP.* (Participant 52)

*We are allowed to remotely discharge people by PaCCS [hear and treat pathway]. So why can I not do it with an ECSW [emergency care support worker] colleague who is actually in the room doing an assessment? They’re taking pictures of ECGs, I can see exactly what they’ve done*. (Participant 42)

*It all relies on us being contacted in the first place*. (Participant 6)

*I do look at crews’ paperwork, ... I look through and just see if people have been on scene a little while or see what the job says it’s going to be and then if I think it’s appropriate for UCR [urgent community response] or anything like that, I’ll send the crew a message*. (Participant 42)

A significant theme from analysis of transcripts was the need for a cultural shift towards normalisation of ECAL use. Participants believed that ambulance clinicians were not accustomed to routine collaborative decision making outside the confines of a ‘crew’. Some were seen as protective of their perceived autonomy, while others seemed to act as if they were seeking a shift of clinical responsibility onto the APP. Participants stressed that ECALs should be viewed culturally as a collaborative process, where both parties can make valuable contributions.

*People see it as a process that they have to go through to discharge a patient rather than, you know, a truly sort of two-way conversation around a decision that they’re making, I think it’s probably more normalised in hospital because it’s less formal.* (Participant 27)

Positive experiences reinforce engagement, and some participants believed that when NQPs who were previously mandated to use ECALs progressed to be classed as experienced paramedics, they would continue to engage due to their familiarity with the inherent benefits. Equally, however, it was stressed that negative experiences would quickly lead to disengagement. ECAL interactions need to be mutually respectful of the individuals involved. Both paternalistic approaches from APPs and defensive stances from crews were cited as examples of poor experiences. APPs must be able to trust what crews are telling them, and crews need to know that APPs are providing them with accurate and appropriate advice. Additionally, the knowledge, experience and risk tolerance of clinicians on both sides of an ECAL were also recognised as influencing its effectiveness.

*I think it only takes one bad call. You know they only need to get someone on a bad day who talks rudely to the crew and makes them feel a bit stupid or uncomfortable. They won’t ring again for a really long time.* (Participant 2)

*It’s got to be a friendly, supportive, educational interaction from the APP hub’s point of view, which apparently isn’t always the perception. But, equally, you’ve got to be happy to receive feedback, change your plan and have an open, honest discussion if you’re asking for clinical advice. I think both of those things have got to be true for it to be a useful interaction that’s going to add any benefit*. (Participant 23)

To address technological and organisational limitations, participants proposed improving the interoperability of health and social care provider information systems, together with consistency in their capabilities across the region and perhaps increased scope for referral to secondary care. This was envisioned to make care navigation less complicated and more efficient. While some advocated the use of video links to conduct ECALs to enable more accurate patient assessment, others felt that this would have limited or no benefit. To address the cultural and relational limitations, alongside an increasingly proactive approach, bi-directional feedback on ECAL interactions and raised awareness of hub capabilities were proposed to increase engagement.

A few participants had experience of two local initiatives that perhaps addressed some of the limitations of other hubs’ activities. These were integrated care hubs that co-located SECAmb APPs, ED consultants and clinicians from community, frailty and urgent treatment centre teams to enable multi-professional urgent care delivery. With variations, these hubs reviewed all calls received by SECAmb in the locality, proactively contacted patients or crews on scene and also responded to crew requests for ECALs. They had access to the various teams’ clinical records and could directly accept appropriate community referrals. This structured triage and supervisory approach was praised by those that had experienced it, who cited improved utilisation of alternative pathways and shared learning as benefits.

*You sit with the community team and someone from the hospital who have got access to all the information on the patient and can book GP appointments in the ED, can send the community teams out, maybe not that day, maybe the next day, and patients can self-convey to a lot of these appointments as well.* (Participant 44)

*I learned a lot from them, but I can tell they were learning off each other, every different person in that room was learning off of everyone else ... how they were thinking*. (Participant 1)

## Discussion

Our findings suggest that clinicians involved with an RDMS system believe that it benefits both patient safety and appropriate care delivery in a ‘see and treat’ response model. However, literature reviews addressing this topic have concluded that there is an absence of empirical evidence to determine whether RDMS is clinically effective or cost effective (Janerka et al., 2023; Kim et al., 2020; NICE, 2018). Further evaluations of patient benefit and economic efficiency are certainly required in this field.

Perceived benefits for patient safety and clinician development were reported by Jackson & Jones (2012) in their mixed-methods study of RDMS, provided by advanced paramedics in the North West Ambulance Service NHS Trust (NWAS). They also identified a reluctance in experienced paramedics to utilise RDMS, alongside higher engagement from university-educated paramedics, which may mirror our findings to some degree. Additionally, our participants’ confirmation of the APP role as ideally suited to provide RDMS is in accordance with qualitative work in Canada that emphasised the value of shared culture and common lived experience in enabling remote collaborative practice (Armour et al., 2022).

Digital siloing of health and social care providers is well recognised as a significant barrier to the provision of integrated holistic urgent care (NHS England, 2019; The King’s Fund, 2022). Unfortunately, our participants also identified some internal technological and governance limitations to effective care navigation, in the recording of hub activity and in securing onward referral. An alignment of urgent care provider capabilities, coupled with a good working knowledge of these among ambulance clinicians, was also proposed by NHS England (2019) to benefit joined-up care delivery. Our participants’ responses seem to indicate that significant variations in capability and capacity may not always be accurately described by directories of services, rendering local knowledge important. These issues highlight that interoperability of providers is not solely a digital consideration, and that interprofessional understanding and establishing an enabling infrastructure are equally important (The King’s Fund, 2022). The integrated care hubs being trialled in the Trust, with co-located clinicians from key providers, may be a good example of how integrating databases and staff, when coupled with organisational commitment, may address many of the barriers to appropriate urgent care delivery identified in this study and elsewhere.

Proactive intervention in incidents was another feature of integrated care hubs that was highlighted as a potential development by our participants to benefit effectiveness, as currently limited engagement from clinicians was perceived to limit hub impact. Despite positive perceptions of RDMS among paramedics being reported in the literature (Armour et al., 2022; Gilligan et al., 2018; Jackson & Jones, 2012), this study suggests that remote collaboration may not yet be viewed culturally as standard procedure. Ambulance clinicians are used to practising in relative isolation and may be protective of their perceived autonomy when approached proactively (Hill & Eaton, 2023). Given that negative experiences of ECAL may have greater impact on engagement than positive ones, our participants stressed the importance of honest collaborative communications, including feedback on the ECAL itself, to maximise RDMS effectiveness. Demonstrating benefit was then anticipated to influence culture.

### Limitations

Despite being a stated aim of the study, the perspectives of clinicians who rarely or never engage with ECALs were not collected. Although the limitations discussed in the study may identify some of the barriers to engagement, these perspectives may have provided further valuable insight. The variation in online meeting participant numbers may have influenced the narratives provided (Holloway & Galvin, 2016). Additionally, combining clinicians that access RDMS and senior colleagues that provide it in meetings may have introduced a power dynamic potentially biasing narratives (Holloway & Galvin, 2016). However, our participants appeared to provide honest reflections, and all were respectful of other contributors’ perspectives. The study was limited to a single ambulance RDMS system and there will be considerable variations in these systems internationally. It is anticipated, though, that many findings will be generalisable to other pre-hospital workforces that employ similar operational models. Finally, it may be worth noting that this is a cross-sectional study of an evolving phenomenon and clinician perspectives are likely to similarly evolve.

## Conclusion

This study suggests that RDMS is perceived as beneficial to patient safety and appropriate care delivery, and that APPs familiar with their region and with the clinicians on scene are well suited to provide this support. Improving the consistency and digital interoperability of community care pathways may permit more effective navigation of care by ambulance clinicians, but further interprofessional collaboration may additionally enhance the integration of these services. Collaborative decision making requires honest and open interaction to be effective, and demonstrating its value may lead to RDMS being more widely accepted as standard clinical practice.

## Acknowledgements

Sincere thanks to all participants for taking the time to contribute their perspectives to this study.

## Guarantor statement

PEW conceived and designed this study, collected and analysed its data and composed the manuscript for publication. PEW acts as the guarantor for this article.

## Conflict of interest

None declared.

## Ethics

Health Research Approval was provided, IRAS 327074. As a study solely involving data collection from consenting NHS staff, NHS ethics approval was not required. Anonymised Trust Research and Development Group approval was also provided prior to study initiation.

## Funding

College of Paramedics Small Research Grant.

## References

[bibr_1] Abrashkin K. A. Poku A. Ramjit A. et al (2019). Community paramedics treat high acuity conditions in the home: A prospective observational study. *BMJ Supportive & Palliative Care*, 14(e1), e683–e690. https://doi.org/10.1136/bmjspcare-2018-001746. 10.1136/bmjspcare-2018-001746 30948443

[bibr_2] Armour R. & Helmer J. (2021). Paramedic-delivered teleconsultations: A scoping review. *Australasian Journal of Paramedicine*, 18, 1–7. https://doi.org/10.33151/ajp.18.882.

[bibr_3] Armour R. Helmer J. & Tallon J. (2022). Paramedic-delivered teleconsultations: A grounded theory study. *Canadian Journal of Emergency Medicine*, 24(2), 167–173. https://doi.org/10.1007/s43678-021-00224-6. 34874528 10.1007/s43678-021-00224-6 PMC8904334

[bibr_4] Association of Ambulance Chief Executives (AACE). (2020). The ambulance response to the COVID-19 pandemic: What went well and how do we sustain the benefits? https://aace.org.uk/wp-content/uploads/2020/08/Ambulance-Response-COVID-19-What-went-well-Final-6th-August-2020.pdf.

[bibr_5] Barrett J. W. Williams J. Skene S. S. et al (2023). Head injury in older adults presenting to the ambulance service: Who do we convey to the emergency department, and what clinical variables are associated with an intracranial bleed? A retrospective case-control study. *Scandinavian Journal of Trauma, Resuscitation and Emergency Medicine*, 31(1), 65. https://doi.org/10.1186/s13049-023-01138-1. 37908011 10.1186/s13049-023-01138-1 PMC10619243

[bibr_6] Braun V. & Clarke V. (2022). Conceptual and design thinking for thematic analysis. *Qualitative Psychology*, 9(1), 3. https://doi.org/10.1037/qup0000196.

[bibr_7] Gilligan P. Bennett A. Houlihan A. et al (2018). *The doctor can see you now: A key stakeholder study into the acceptability of ambulance based telemedicine*. Royal College of Surgeons in Ireland. https://repository.rcsi.com/articles/journal_contribution/The_Doctor_Can_See_You_Now_A_Key_Stakeholder_Study_Into_The_Acceptability_Of_Ambulance_Based_Telemedicine_/10767731. 30518784

[bibr_8] Hill L. & Eaton G. (2023). Exploring paramedic professional identity. *British Paramedic Journal*, 8(3), 42–51. https://doi.org/10.29045/14784726.2023.12.8.3.42. 10.29045/14784726.2023.12.8.3.42 PMC10690486 38046791

[bibr_9] Holloway I. & Galvin K. (2016). *Qualitative research in nursing and healthcare*. John Wiley & Sons.

[bibr_10] Jackson M. & Jones C. (2012). Kerbside consultations: Advice from the advanced paramedic to the frontline. *Journal of Paramedic Practice*, 4(9), 526–532. https://doi.org/10.12968/jpar.2012.4.9.526.

[bibr_11] Janerka C. Leslie G. D. Mellan M. et al (2023). Review article: Prehospital telehealth for emergency care: A scoping review. *Emergency Medicine Australasia*, 35(4), 540–552. https://doi.org/10.1111/1742-6723.14224. 37102271 10.1111/1742-6723.14224

[bibr_12] Kim Y. Groombridge C. Romero L. et al (2020). Decision support capabilities of telemedicine in emergency prehospital care: Systematic review. *Journal of Medical Internet Research*, 22(12), e18959. https://doi.org/10.2196/18959. 33289672 10.2196/18959 PMC7755537

[bibr_13] Maxwell J. A. (2012). *A realist approach for qualitative research*. Sage.

[bibr_14] National Institute for Health and Care Excellence (NICE). (2018). *Paramedic remote support. Emergency and acute medical care in over 16s: Service delivery and organisation. NICE guideline 94 evidence review*. NICE. https://www.nice.org.uk/guidance/ng94/evidence/4paramedic-remote-support-pdf-4788818465. 33270407

[bibr_15] NHS England. (2019). *Planning to safely reduce avoidable conveyance. Ambulance Improvement Programme*. NHE England and NHS Improvement. https://www.england.nhs.uk/wp-content/uploads/2019/09/planning-to-safetly-reduce-avoidable-conveyance-v4.0.pdf.

[bibr_16] O’Hara R. Johnson M. Hirst E. et al (2014). A qualitative study of decision-making and safety in ambulance service transitions. *Health Services and Delivery Research*, 2(56). https://www.ncbi.nlm.nih.gov/books/NBK269187/. 25642577

[bibr_17] Rapport F. Hogden A. Faris M. et al (2018). *Qualitative research in healthcare: Modern methods, clear translation: A white paper*. Australian Institute of Health Innovation, Macquarie University, Sydney, Australia.

[bibr_18] The King’s Fund. (2022). *Interoperability is more than technology. The role of culture and leadership in joined-up care*. https://www.kingsfund.org.uk/insight-and-analysis/reports/digital-interoperability-technology.

[bibr_19] University of Auckland. (2024). *Thematic analysis*. University of Auckland. https://www.thematicanalysis.net.

[bibr_20] Yardley L. (2000). *Psychology and Health*, 15(2), 215–228. http://dx.doi.org/10.1080/08870440008400302.

